# Pregnancy and fetal outcomes after Glatiramer Acetate exposure in patients with multiple sclerosis: a prospective observational multicentric study

**DOI:** 10.1186/1471-2377-12-124

**Published:** 2012-10-22

**Authors:** Marta Giannini, Emilio Portaccio, Angelo Ghezzi, Bahia Hakiki, Luisa Pastò, Lorenzo Razzolini, Elisa Piscolla, Laura De Giglio, Carlo Pozzilli, Damiano Paolicelli, Maria Trojano, Maria Giovanna Marrosu, Francesco Patti, Loredana La Mantia, Gianluigi Mancardi, Claudio Solaro, Rocco Totaro, Maria Rosaria Tola, Giovanna De Luca, Alessandra Lugaresi, Lucia Moiola, Vittorio Martinelli, Giancarlo Comi, Maria Pia Amato

**Affiliations:** 1 Department of Neurology, University of Florence, Florence, Italy; 2 Multiple Sclerosis Center, S Antonio Abate Hospital, Gallarate, Italy; 3 Multiple Sclerosis Center, S Andrea Hospital, La Sapienza University, Rome, Italy; 4 Department of Neurology, University of Bari, Bari, Italy; 5 Multiple Sclerosis Centre Department of Neurology, University of Cagliari, Cagliari, Italy; 6 Multiple Sclerosis Centre, University of Catania, Catania, Italy; 7 MS Center, C Besta National Neurological Institute, Milan, Italy; 8 Department of Neurology, University of Genova, Genova, Italy; 9 Department of Neurology, ASL3 Genovese, Genova, Italy; 10 Department of Neurology, University of L’Aquila, L’Aquila, Italy; 11 Department of Neuroscience, University of Ferraral, Ferrara, Italy; 12 Department of Neuroscience, University of Ferrara, Ferrara, Italy; 13 Department of Neurology, Scientific Institute H. San Raffaele University of Milan, Milan, Italy; 14 Don Carlo Gnocchi Foundation ONLUS

**Keywords:** Glatiramer acetate, Multiple sclerosis, Pregnancy, Pregnancy outcome, In utero exposure

## Abstract

**Background:**

Only few studies have assessed safety of in utero exposure to glatiramer acetate (GA). Following a previous study assessing the safety of interferon beta (IFNB) pregnancy exposure in multiple sclerosis (MS), we aimed to assess pregnancy and fetal outcomes after in utero exposure to GA, using the same dataset, with a specific focus on the risk of spontaneous abortion.

**Materials and methods:**

We recruited MS patients, prospectively followed-up in 21 Italian MS Centres, for whom a pregnancy was recorded in the period 2002–2008. Patients were divided into 2 groups: drug-exposed pregnancies (EP: suspension of the drug less than 4 weeks from conception); non-exposed pregnancies (NEP: suspension of the drug at least 4 weeks from conception or never treated pregnancies). All the patients were administered a structured interview which gathered detailed information on pregnancy course and outcomes, as well as on possible confounders. Multivariate logistic and linear models were used for treatment comparisons.

**Results:**

Data on 423 pregnancies were collected, 17 were classified as EP to GA, 88 as EP to IFNB, 318 as NEP. Pregnancies resulted in 16 live births in the GA EP, 75 live births in the IFNB EP, 295 live births in the NEP. GA exposure was not significantly associated with an increased risk of spontaneous abortion (OR = 0.44;95% CI 0.044-4.51;p = 0.49). Mean birth weight and length were not significantly different in pregnancies exposed to GA than in non exposed pregnancies (p = 0.751). The frequency of preterm delivery, observed in 4 subjects exposed to GA (25% of full term deliveries), was not significantly higher in pregnancies exposed to GA than in those non exposed (p > 0.735). These findings were confirmed in the multivariate analysis. There were neither major complications nor malformations after GA exposure.

**Conclusions:**

Data in our cohort show that mother’s GA exposure is not associated with a higher frequency of spontaneous abortion, neither other negative pregnancy and fetal outcomes. Our findings point to the safety of in utero GA exposure and can support neurologists in the therapeutic counselling of MS women planning a pregnancy.

## Introduction

Since Multiple Sclerosis (MS) predominantly affects women in childbearing phase of their lifes, the issue on the tolerability and safety of disease-modifying therapy (DMT) use in pregnant relapsing-remitting (RR) MS patients has become increasingly important. This concept is especially prominent in subjects with highly active MS, in which the discontinuation of DMT in case of pregnancy planning can expose the patients to the risk of relapses. Over the past few years several studies have suggested that exposure to Interferon-β (IFNB) therapies during early pregnancy period is safe [1-4]. As for glatiramer acetate (GA) data in humans are rather limited. To date, GA is classified by US FDA as a Category B drug in relation to pregnancy, due to the absence of foetal risk in animal studies [5]. However few studies suggested the absence of adverse outcomes from GA exposure during the early pregnancy [4,6-8]. In particular, a prospective observational controlled cohort study on 31 pregnancies exposed to GA found neither drug-specific adverse pregnancy outcomes nor birth defects compared to an MS and non-MS control groups [6]. Moreover, two smaller uncontrolled studies reported a preliminary experience on continuous exposure of GA throughout the whole course of pregnancy [9,10]. Neither drug-related obstetric/neonatal complications nor malformations, were reported in the pregnancies exposed to GA. The Authors also preliminarly suggested a protective role of continuos exposure to GA during pregnancy against the risk of relapses in the post-partum period.

In a previous multicentric, prospective study we collected pregnancies followed-up in the main Italian MS centers and addressed the issue of IFNB exposure. In the same study we also gathered detailed information on pregnancies exposed to GA [1]. The objective of this further analysis was to determine the safety of in utero exposure to GA in terms of pregnancy and foetal outcomes as well as developmental abnormalities of the babies after birth.

## Methods

Recruitment of the patients begun in January 2002 and ended in January 2008. In this period, all pregnancies beginning in MS patients diagnosed according to MacDonald’s criteria [11] and referred to the participating centers were identified and tracked over the whole gestational period. The 21 participating sites represented the main Italian MS Centers located throughout the entire country. In each center the patients were regularly followed-up every six months and in the case of relapse. As described elsewhere [1], clinical and therapeutic data were gathered by the neurologist using a standardized information form. Within six months after the delivery, the neurologist administered a semi-structured interview to each patient dealing with pregnancy outcomes and potential confounders. A specific section gathered information on the pregnancies occurring during the study period, focusing on *in utero* exposure to toxins, smoke, alcohol, pharmacological therapies and timing of suspension in relationship with conception. Substance exposure status was defined as “exposed “ (any exposure to any substance in any trimester) and “never exposed” (no substance exposure). Maternal smoking status was defined as “never smoked” (no smoking in any trimester) and “smoked” (smoking in any trimester). Alcohol exposure status was defined as “exposed” (drinking more than one unit per day), and “not-exposed” (drinking less than one unit per day). Conception was determined as 14 days after the mother’s last menstrual period. Follow-up data of the babies were also gathered and up-dated every six months, following a specific section of the questionnaire for the parents that investigated major developmental problems and malformations detected following birth. In case of problems, the baby’s clinical charts were reviewed.

Patients were divided into two groups: those who had discontinued the GA less than four weeks from conception (exposed), and those who had discontinued the drug at least four weeks from the conception or who had never been treated with DMTs (not-exposed). Data on spontaneous abortion (occurring before 22 completed weeks of gestation) and other outcomes (in particular, weight and length of the baby, pre-term delivery, cesarean delivery) were compared between the exposed and not-exposed groups. The study was approved by the ethic committee of the University of Florence and a written consent was obtained from all patients.

### Statistical analysis

Spontaneous abortion, birth-weight and birth-length, pre-term delivery (<37 weeks) and caesarean delivery were analyzed. Group comparisons was assessed with Pearson’s χ [2] and Mann–Whitney U tests or analysis of variance (ANOVA) with Bonferroni correction for multiple comparisons, when appropriate.

Multivariate logistic and linear models were used for treatment comparisons, including as confounders age at conception, educational level (as an indicator of the socioeconomic status), disease duration, Expanded Disability Status Scale [12], previous pregnancies and abortions (yes vs no), smoking, alcohol and substance exposure during pregnancy (yes vs no), gestational age, caesarean delivery, gender of the baby. In the multivariate analyses pregnancies exposed to IFNB or previously treated with IFNB were excluded. Results are expressed as odds ratios (ORs) or estimated means, and 95% confidence intervals (CIs). P-values less than 0.05 were considered significant.

We used the SPSS 18.0 running on Windows (SPSS, Chicago, IL, USA).

## Results

### Study sample

**Figure 1:**
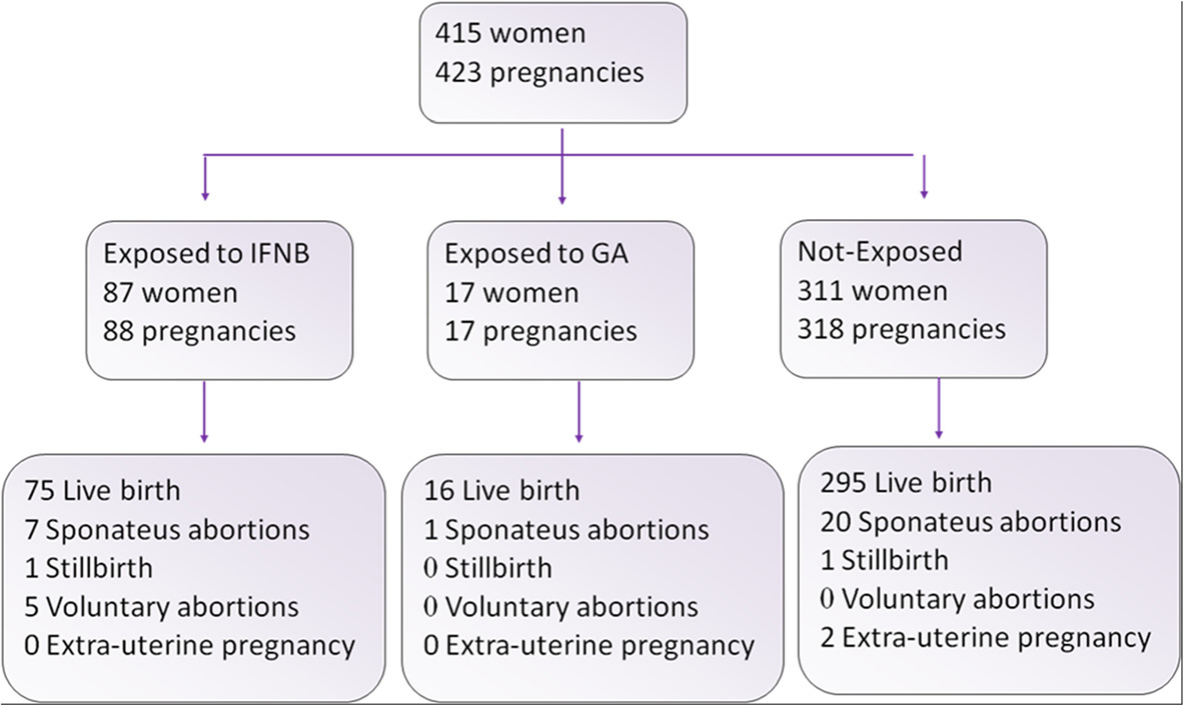
Flow-chart of the pregnancies enrolled in the study GA: glatiramer acetate IFNB: Interferon-β.

A total of 423 pregnancies were tracked in 415 women. The last pregnancy included took place on January 2008 (Figure. 1). The median follow-up period after the end of pregnancy was 2.1 years. No woman was lost to the follow-up. Forty (9.5%) women received glucocorticoid treatment for relapses during pregnancy (58.8% during the first, 32.4% during the second and 8.8% during the third trimester). All glucocorticoid treatments during pregnancy occurred in the non-exposed patients, although the difference was not statistically significant (p = 0.173). No patient received intravenous immunoglobulins during the pregnancy.

#### Abortion

Full data on pregnancy and delivery were available for 423 pregnancies in 415 women (Table 1). Eighty-eight pregnancies were exposed to IFNB (10 to Betaferon, 21 to Rebif 22, 22 to Rebif 44, 35 to Avonex) for a mean duration of exposure of 4.6 ± 5.8 weeks (range −4 - 41) and 17 pregnancies were exposed to GA for a mean duration of exposure of 4.9 ± 2.8 weeks (range 2–12).

**Table 1:** Characteristics of the study sample

	Exposed to GA * (#17)	**Non exposed to GA (# 202)**	**p**
Age at conception, mean (SD) years	31.8 (3.7)	31.8 (5.1)	0.987
Education, mean (SD) years	13.3 (3.8)	13.2 (3.6)	0.937
Disease duration, mean (SD) years	8.3 (4.6)	6.8 (5.5)	0.308
EDSS at conception, mean (SD)	2.0 (1.4)	1.3 (0.8)	0.002
Previous abortion (#,%)	1 (5.9)	18 (8.9)	0.670
Previous pregnancy (#,%)	3 (17.6)	33 (16.4)	0.845
Smoking during pregnancy (#,%)	2 (11.8)	15 (7.5)	0.525

*Mean duration of exposure: 4.9 ± 2.8 weeks.

GA: Glatiramer Acetate; SD: Standard Deviations; EDSS: Expanded Disability Status Scale.

We observed 386 full-term deliveries (217 males, 169 females), 75 in exposed to IFNB, 16 in exposed to GA and 295 in non-exposed subjects. There were eight twin pregnancies. Epidural analgesia was given to 70 (18.1%) women. Spontaneous abortions were observed in 28 pregnancies, one (5.9%) in exposed to GA after a 12 weeks of gestation, seven (7.9%) in exposed to IFNB after a mean of 8.2 ± 1.6 weeks of gestation, and 20 (6.3%) in non-exposed patients, after a mean of 8.5 ± 2.3 weeks. Moreover, we observed two still births (one in IFNB exposed group and one in non-exposed group), two extra-uterine pregnancies (both in the non-exposed group) and five voluntary abortions, all in the IFNB exposed group.

The risk of spontaneous abortion after GA exposure was assessed comparing exposed pregnancies (#17) with pregnancies occurring in patients previously treated with GA or never treated with other DMDs (#202), excluding patients who had received IFNBs (Table 1). Patients exposed to GA did not differ from patients non-exposed in terms of demographic and clinic characteristics, with the exception of EDSS score that was significantly higher in patients exposed to GA (Table 1).

In the multivariate analysis, spontaneous abortion was related only with higher age at conception and previous pregnancies (Table 2). GA exposure did not increase the risk of spontaneous abortion (OR = 0.44; 95% CI 0.04-4.51; p = 0.492).

**Table 2:** Significant predictors of spontaneous abortion, preterm delivery, caesarean delivery

		**OR**	**95% CI**	**p**
Spontaneous abortion*				
	Higher age at conception	1.23	1.04 - 1.46	0.018
	Previous pregnancies	3.12	1.01 - 9.69	0.048
Preterm delivery*				
	Caesarean delivery	0.22	0.10 – 0.49	< 0.001
Caesarean delivery*				
	Gestational age	0.72	0.62 – 0.85	< 0.001
	Education	0.90	0.81 – 0.98	0.022

* Other possible predictors included in the models (age at conception, educational level, disease duration, Expanded Disability Status Scale, previous pregnancies and abortions, smoking, alcohol glucocorticoid and substance exposure during pregnancy, gestational age, caesarean delivery, gender of the baby) were not significantly associated with the dependent variable (p > 0.11).

OR: Odd Ratio; CI: Confidence Interval.

#### Preterm and caesarean delivery

Mean gestational age was 38.6 ± 2.6 weeks in pregnancies exposed to GA, 37.8 ± 2.1 in those exposed to IFNB, and 38.5 ± 2.4 in those non epxosed. Gestational age was significantly lower after exposure to IFNB than in non exposed (p = 0.048). There was no difference between pregancies exposed to GA and either those exposed to IFNB or non exposed (p > 0.735).

Preterm delivery was observed in 4 subjects exposed to GA (25% of full term deliveries), 25 exposed to IFNB (32.8% of full term deliveries), and 58 in non exposed (20.1% of full term deliveries).

Caesarean delivery was observed in 7 subjects exposed to GA (43.8% of full term deliveries), 34 exposed to IFNB (44.7% of full term deliveries), and 130 non exposed (45.1% of full term deliveries).

As for the analysis on spontaneous abortion, patients who had received IFNBs were excluded. In the multivariate analysis, caesarean delivery was related only to preterm delivery, whereas lower gestational age and lower education were associated with caesarean delivery (Table 2).

#### Birth weight and length

Mean birthweight was 3357 ± 616 gr in pregnancies exposed to GA, 3010 ± 513 gr in those exposed to IFNB, and 3209 ± 488 in those non exposed. Mean birthweight was significantly lower after exposure to IFNB than in other two groups (p < 0.036). There was no difference between pregancies exposed to GA and those non exposed (p = 0.751).

Mean birthlength was 50.1 ± 3.1 cm in pregnancies exposed to GA, 48.7 ± 3.4 in those exposed to IFNB, and 49.9 ± 3.2 in those non exposed. Mean birth length was significantly lower after exposure to IFNB than in non exposed (p = 0.014). There was no difference between pregancies exposed to GA and either those exposed to IFNB or non exposed (p > 0.360).

In the multivariate analysis (excluding patients who had received IFNBs), an older age at disease onset, preterm and caesarean delivery were related to a lower weight, whereas only preterm delivery was associated to a lower length of the babies (Table 3).

**Table 3:** Significant predictors of birth weight and length

	**Beta**	**p**
Birthweight*		
Older age at disease onset	−0.188	0.012
Preterm delivery	−0.331	< 0.001
Caesarean delivery	−0.233	0.003
Birthlength*		
Preterm delivery	−0.307	< 0.001

* Other possible predictors included in the models (age at conception, educational level, disease duration, Expanded Disability Status Scale, previous pregnancies and abortions, smoking, alcohol glucocorticoid and substance exposure during pregnancy, gestational age, caesarean delivery, gender of the baby) were not significantly associated with the dependent variable (p > 0.12).

#### Pregnancy complications and follow-up of the babies

Maternal complications occurred in 4 out of 16 (25%) full term deliveries in the GA exposed pregnancies and in 28 out of 190 (15%) in the non exposed group (Table 4). The most frequent complication was threatened abortion in the non-exposed group. In exposed women we observed one case of placental abnormality, one case of eclampsia, one case of cholestasis and one case of headache. No fetal complication was observed in pregnancies exposed to GA. In the non exposed group, the most frequent fetal complication was minor respiratory distress, not requiring intensive care (Table 4). Finally, in a median follow-up period of 2.1 years, we observed two developmental abnormalities (mild speech disorders), both in the non exposed group.

**Table 4:** Maternal and fetal complications in exposed and non-exposed pregnancies

	**GA-Exposed pregnancies (#16)**	**Non-exposed pregnancies (#190)**
Maternal complications	1 placental detachment	8 threatened abortion
	1 pre-eclampsia/eclampsia	4 placentar abnormalities
	1 headache	5 gestational diabetes
	1 cholestasis at VI month	3 pre-eclampsia/eclampsia
		2 kidney colic
		2 varicella infection
		1 minor infection
		1 uterine atony
		1 meningism
		1 metrorrhagia
Fetal complications	0	4 respiratory distress
		2 jaundice
		1 acute fetal distress
		1 intrauterine growth retardation
		1 hip dysplasia
		1 dehydration
		1 anemia requiring transfusion
		1 renal pelvis distension

GA: glatiramer acetate.

## Discussion

To date the information on pregnancy and fetal outcomes after in-utero exposure to GA in patients with MS is limited. The main data on the safety of GA is from the manufacturer’s post-marketing surveillance, that suggests no increased risk in terms of spontaneous abortion and other outcomes. Indeed the reported miscarriage rate (17%) is similar to that observed in the general population [7].

With regard the experience of Italian centers routine practice was to discontinue DMDs before a pregnancy program and to advise preventing pregnancy while on therapy. However, in a previous multicentric, prospective study that was focused on the issue of IFNB exposure, we observed 115 unplanned pregnancies occurred during DMD treatment (88 exposed to IFNB, 17 to GA). As for GA, in the present study we found no significant association between drug exposure and increased risk of spontaneous abortion, after an average exposure of approximately 4 weeks. Moreover, the risk of spontaneous abortion was comparable to that of the general population.

Similar findings were reported by Weber-Schoendorfer et al., in a prospective observational cohort study [6], assessing the outcomes of 69 pregnancies exposed to IFNB and 31 exposed to GA (the latter with a median duration of exposure of 6.9 weeks).

As for other pregnancy and fetal outcomes, in our study GA exposure was associated with neither a higher risk of preterm and caesarean delivery nor a lower weight and length of the babies. It has to be noted that lower birth-weight and –lenght resulted to be related with the exposure to IFNB. This difference was observed also by Weber-Schoendorfer and co-workers. This result may be due, at least in part, by the different mechanism of action of the drugs. Although IFNBs are macromolecules that do not easily pass the placenta, it is known that cytokine imbalance may affect pregnancy outcomes [13-15].

Furthermore no fetal complication was observed in pregnancies exposed to GA and , in a median follow-up period of 2.1 years, we did not observe developmental abnormalities in the GA exposed group. Probably a low risk of GA teratogenicity may be due to its mechanism of action [16].

In interpreting the study results, a few possible limitations should be taken into account. The sample size of GA-exposed pregnancies is small. Moreover, we cannot exclude the occurrence of recall bias, even if women were prospectively followed up and the interview was performed soon after the delivery.

Our study focused on the effects of GA after an average exposure of approximately 4 weeks, and the consequences of longer duration of exposure cannot be assessed. However, two smaller uncontrolled studies and a another recent study performed on a larger population of patients reported the safety of continuous exposure of GA for at least eight weeks of gestation [4,9,10]. Neither drug-related obstetric/neonatal complications nor malformations were observed in the pregnancies exposed to GA.

Despite these limitations, taken as a whole our findings point to the safety of GA utero exposure in terms of the risk of spontaneous abortion and the main maternal and fetal outcomes. With respect to what observed after IFNB exposure, GA exposure did not affect gestational age, birth-weight and –length, possibly resulting in a better safety profile. Our study can assist the neurologist facing the issue of pregnancy planning in MS patients. In particular, in subjects with high disease activity and high risk of post-partum relapses, prosecution of GA treatment may be considered.

## Abbreviations

MS: Multiple sclerosis; IFNB: Interferon-β; GA: Glatiramer acetate; EP: Drug-exposed pregnancies; NEP: Non-exposed pregnancies; DMT: Disease-modifying therapy; RR: Relapsing-remitting; ORs: Odds ratios; CIs: Confidence intervals; SD: Standard Deviations; EDSS: Expanded Disability Status Scale.

## Authors’ contributions

**MG**, Drafting/revising the manuscript Study concept or design Analysis or interpretation of dataAcquisition of data **EP**, Drafting/revising the manuscript Study concept or design Analysis or interpretation of data Acquisition of data Statistical analysis Study supervision **AG,** Drafting/revising the manuscript Study concept or design Acquisition of data **BH**, Study concept or design Acquisition of data **LP**, Study concept or design Acquisition of data **LR,** Study concept or design Acquisition of data **EP,** Acquisition of data **LDG**, Analysis or interpretation of data Acquisition of data **CP**, Drafting/revising the manuscript Study supervision **DP**, Analysis or interpretation of data Acquisition of data **MT,** Drafting/revising the manuscript Study concept or design Acquisition of data **MGM**, Drafting/revising the manuscript Acquisition of data **FP**, Drafting/revising the manuscript Study concept or design Analysis or interpretation of data Acquisition of data **LLM**, Drafting/revising the manuscript Acquisition of data **GM**, Study concept or design Acquisition of data **CS**, Analysis or interpretation of data Study supervision **RT**, Drafting/revising the manuscript Acquisition of data **MRT**, Drafting/revising the manuscript Study concept or designAcquisition of data**DT**, Analysis or interpretation of data Acquisition of data **AL**, Drafting/revising the manuscript Analysis or interpretation of data Acquisition of data **LM,** Analysis or interpretation of data Acquisition of data **VM,** Drafting/revising the manuscript Analysis or interpretation of data Acquisition of data **GC**, Drafting/revising the manuscript Study supervision **MPA**, Drafting/revising the manuscript Study concept or design Analysis or interpretation of data Study supervision. All authors read and approved the final manuscript.

## Disclosures

**Dr. Giannini** has received compensation from Biogen Idec.

**Dr. Portaccio** serves on a scientific advisory board for Biogen Idec and receives research support from Merck Serono, Biogen Idec, Bayer Schering Pharma, and sanofi-aventis.

**Dr. Ghezzi** serves on scientific advisory boards for Merck Serono and Teva Pharmaceutical Industries Ltd.; has received speaker honoraria from Merck Serono, Biogen Idec, Bayer Schering Pharma, and Novartis; serves as a consultant for Novartis; and receives research support from

Sanofi-aventis, Biogen Idec, and Merck Serono.

**Dr. Hakiki** receives research support from Novartis and Merck Serono; received funding for travel from Biogen, Sanofi, Novartis, Bayer, Merck Serono.

**Dr. Pastò** has received compensation from Biogen Idec.

**Dr. Razzolini** has received funding for travel and research support from Novartis.

**Dr. Piscolla** has nothing to disclose.

**Dr. Pozzilli** serves on scientific advisory boards for and has received speaker honoraria from Novartis, Merck Serono, Biogen Idec, Bayer Schering Pharma, and sanofi-aventis.

**Dr. De Giglio** reports no disclosures.

**Dr. Paolicelli** serves as a consultant for Merck Serono and Bayer Schering Pharma.

**Dr. Trojano** has received speaker honoraria from Merck Serono, Bayer Schering Pharma, sanofi-aventis, and Biogen Idec; and has received research support from Biogen Idec and Merck Serono.

**Dr. Marrosu** serves on scientific advisory boards for Merck Serono, Biogen Idec, and Bayer Schering Pharma; has received funding for travel from Biogen Idec, Merck Serono, Bayer Schering Pharma, and sanofi-aventis; serves on the editorial board of *Neurological Sciences*; has received speaker honoraria from Biogen Idec and Merck Serono; and has received research support from

Merck Serono, Biogen Idec, and Fondazione Banco di Sardegna.

**Dr. Patti** has served on scientific advisory boards for Merck Serono, Bayer Schering Pharma, Novartis, and Biogen Idec; has received speaker honoraria from Biogen Idec, Bayer Schering Pharma, sanofi-aventis, and Novartis; and has received research support from the University of Catania and FISM

**Dr. La Mantia** has received funding for travel from Biogen Idec and Bayer Schering Pharma

**Dr. Mancardi** has received funding for travel from Biogen Idec, Merck Serono, and Bayer Schering Pharma; serves on the editorial board of *Neurological Sciences*; and has received speaker honoraria from Biogen Idec and Bayer Schering Pharma.

**Dr. Solaro** reports no disclosures.

**Dr. Totaro** has received honoraria for consultancy or speaking from sanofi-aventis, Biogen Idec, Bayer Schering Pharma, and Merck Serono.

**Dr. Tola** has served on scientific advisory boards for and received speaker honoraria from Biogen Idec, sanofi-aventis, Merck Serono, and Novartis; and has received research support fromsanofi-aventis.

**Dr. Di Tommaso** reports no disclosures.

**Dr. Lugaresi** has served on scientific advisory boards for Biogen Idec, Merck Serono, and Bayer Schering Pharma; has received funding for travel and speaker honoraria from Bayer Schering Pharma, Biogen Idec, Merck Serono, Novartis, Sanofi Aventis, and Teva Pharmaceutical Industries Ltd.; serves as a consultant for Fondazione "Cesare Serono"; and has received research support from Fondazione Italiana Sclerosi Multipla, Bayer Schering Pharma, Biogen Idec, Merck Serono, Sanofi Aventis, Novartis, and AISM (Associazione Italiana Sclerosi Multipla).

**Dr. Moiola** reports no disclosures.

**Dr. Martinelli** has received funding for travel and speaker honoraria from Biogen Idec, Merck Serono, Bayer Schering Pharma, Novartis, and sanofi-aventis; and has served as a consultant to Bayer Schering Pharma, sanofi-aventis, and Teva Pharmaceutical Industries Ltd.

**Dr. Comi** serves on scientific advisory boards for Bayer Schering Pharma, Merck Serono, Teva Pharmaceutical Industries Ltd., sanofi-aventis, Novartis, and Biogen Idec; and has received speaker honoraria from Teva Pharmaceutical Industries Ltd., sanofi-aventis, Serono Symposia International Foundation, Biogen Idec, Merck Serono, Novartis, and Bayer Schering Pharma.

**Dr. Amato** serves on scientific advisory boards for and has received speaker honoraria and research support from Biogen Idec, Merck Serono, Bayer Schering Pharma, and sanofi-aventis; and serves on the editorial board of *BMC Neurology*.

## Pre-publication history

The pre-publication history for this paper can be accessed here:

http://www.biomedcentral.com/1471-2377/12/124/prepub
